# Assessing the Use of Molecular Barcoding and qPCR for Investigating the Ecology of *Prorocentrum minimum* (Dinophyceae), a Harmful Algal Species

**DOI:** 10.3390/microorganisms9030510

**Published:** 2021-02-28

**Authors:** Kate McLennan, Rendy Ruvindy, Martin Ostrowski, Shauna Murray

**Affiliations:** Faculty of Science, University of Technology Sydney, Ultimo, NSW 2007, Australia; kate.mclennan@alumni.uts.edu.au (K.M.); rendy.ruvindy@uts.edu.au (R.R.); martin.ostrowski@uts.edu.au (M.O.)

**Keywords:** *Prorocentrum minimum*, harmful algae, next-generation sequencing

## Abstract

*Prorocentrum minimum* is a species of marine dinoflagellate that occurs worldwide and can be responsible for harmful algal blooms (HABs). Some studies have reported it to produce tetrodotoxin; however, results have been inconsistent. qPCR and molecular barcoding (amplicon sequencing) using high-throughput sequencing have been increasingly applied to quantify HAB species for ecological analyses and monitoring. Here, we isolated a strain of *P. minimum* from eastern Australian waters, where it commonly occurs, and developed and validated a qPCR assay for this species based on a region of ITS rRNA in relation to abundance estimates from the cultured strain as determined using light microscopy. We used this tool to quantify and examine ecological drivers of *P. minimum* in Botany Bay, an estuary in southeast Australia, for over ~14 months in 2016–2017. We compared abundance estimates using qPCR with those obtained using molecular barcoding based on an 18S rRNA amplicon. There was a significant correlation between the abundance estimates from amplicon sequencing and qPCR, but the estimates from light microscopy were not significantly correlated, likely due to the counting method applied. Using amplicon sequencing, ~600 unique actual sequence variants (ASVs) were found, much larger than the known phytoplankton diversity from this region. *P. minimum* abundance in Botany Bay was found to be significantly associated with lower salinities and higher dissolved CO_2_ levels.

## 1. Introduction

In recent decades, there has been an apparent global increase in the range, intensity, and frequency of harmful algal blooms (HABs) linked to a variety of factors, including range expansions, increases in anthropogenic nutrients into coastal water bodies, and increased aquaculture [1,2,3,4,5,6]. *Prorocentrum minimum* is a planktonic marine dinoflagellate that forms HABs and is found commonly in temperate estuarine and coastal waters [7]. *P. minimum* blooms are most common in eutrophic coastal waters of the northern hemisphere; however, they have also been reported in tropical and subtropical regions globally [1,7,8,9,10]. Although few studies have been conducted on *P. minimum* in Australia, it is known to occur in high abundances in some regions, with frequent blooms in the Hawkesbury River in New South Wales (NSW) [11]. In line with the global increase of HABs, *P. minimum* appears to have expanded its geographical range over the last 40 years [1,10,12]. *P. minimum* usually blooms in warm brackish waters that are heavily impacted by excess nutrients, which has led to its presence being used as an indicator of eutrophication in water bodies in the northern hemisphere [1,11,13].

The abundance and even dominance of *P. minimum* in dynamic estuarine and coastal systems may be due to its broad salinity tolerance range of 5–17 PSU [9,14] and broad temperature tolerance range of 3–30 °C [1,15,16]. *P. minimum* typically blooms in low-turbulence environments during periods of high irradiance levels [1]; however, it has been demonstrated that the species can survive complete darkness for extended periods [17], which may allow it to survive in ship ballast waters. *P. minimum* is considered to be a mixotroph, able to supplement its nutrient intake due to feeding on smaller microbes, such as *Cryptomonas* spp., in response to depleted nutrients in the water [7,11,18]. Despite the ability to survive with low nutrients, *P. minimum* preferentially grows in water bodies with high nutrient loadings, typical of eutrophic water bodies [1,9]. *P. minimum* growth has been found to be associated with high inorganic nitrogen (N) and phosphorus (P), strongly linked to anthropogenic sources, such as fertilisers [7]. 

*P. minimum* blooms have been associated with several different marine biotoxins [19,20,21,22]; however, the identities of the compounds and their modes of toxicity are debated. *P. minimum* blooms have shown toxic effects on shellfish, including mortality, poor development, and altered behaviours [1,23,24,25]. Recently, a *P. minimum* bloom has been associated with the neurotoxin tetrodotoxin (TTX) [19,26,27], possibly due to bacterial species associated with *P. minimum* [26,28]. It has been suggested that *P. minimum* toxicity is variable depending on the strain of the species and the environmental conditions under which it is grown [1,26]. Due to incidences of toxin accumulation in shellfish and the impacts on shellfish growth of *P. minimum* toxins, it is an important HAB species to monitor in shellfish-harvesting regions [1,11,23,24,26]. 

Until recently, light microscopy has been the only routine method available to identify and manually count HAB species [29,30,31,32]. However, this method is relatively time-consuming, requires a very high level of taxonomic expertise, and is not able to identify cryptic species, which may appear morphologically indistinguishable from one another despite toxicological differences. For these reasons, alternative methods of monitoring HABs have been developed. Molecular genetic techniques can provide rapid and sensitive HAB monitoring [29,30,33,34]. Two molecular genetic methods used are quantitative polymerase chain reaction (qPCR) and molecular barcoding. Molecular barcoding, also referred to as amplicon sequencing, is becoming invaluable in studying marine ecological assemblages, as it allows for uncultured cells in samples to be identified [29,35,36]. However, due to the existence of variability in the copy numbers of genes among microalgal species, particularly in dinoflagellates [37,38,39], the number of gene copies amplified may not reflect the relative abundance of species in the sample. There is also a bias introduced with the use of broad-range primers, which can lead to certain sequences being preferentially amplified, giving a skewed proportional abundance of target species [29,40]. For this reason, the quantification of dinoflagellate species using amplicon sequencing is uncertain and not accurate when compared with other methods, including qPCR and light microscopy [29,41]. The use of molecular barcoding, which provides an overview of the genetic composition of microbial communities, in conjunction with qPCR, may improve the quantitative assessment of the impact of HAB species in the context of, for example, seasonal changes in the wider microbial community. 

The aim of the study was to develop and assess new molecular genetic approaches to investigate the dynamics, community, and environmental drivers of *P. minimum* in an Australian estuary. To do this, a local isolate of *P. minimum* from Australia was established, and qPCR approaches were designed and tested for the detection and quantification of *P. minimum*. In addition, 18S rRNA amplicon sequences from estuarine water samples were examined to compare the specificity, detection limits, and quantification accuracy of the methods. Environmental samples, including physico-chemical data, were collected monthly for 14 months from 2016 to 2017 from two sites in Botany Bay, an estuary in southeast Australia. Data of the entire microbial community, the abundance of *P. minimum*, and the corresponding physico-chemical variables were examined to assess the factors impacting the presence and abundance of *P. minimum* in an Australian estuary. 

## 2. Materials and Methods 

### 2.1. Sampling Sites

**Figure 1:**
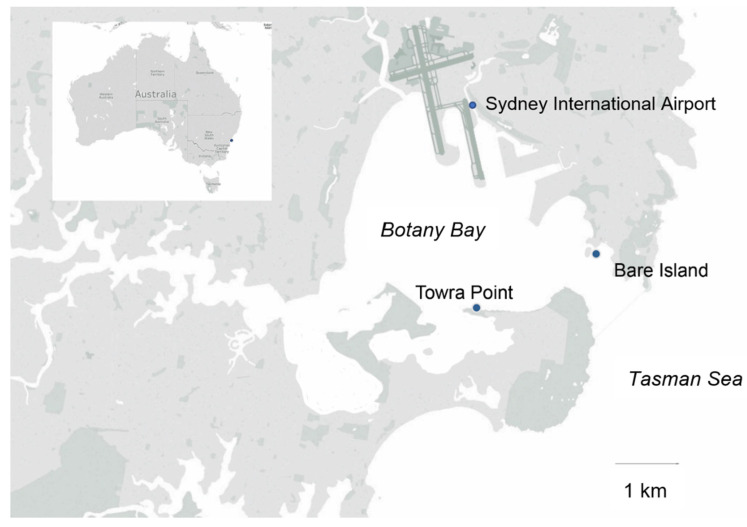
Map of Botany Bay in southeast Australia, highlighting the two sampling points used for the Marine Microbes project, Bare Island and Towra Point.

Fortnightly, phytoplankton samples were collected from two sites, Towra Point (−34.007, 151.19) and Bare Island (−33.992, 151.23), which are both located in Botany Bay, a heavily modified estuary in southeast Australia (Figure 1), as part of the coastal and benthic sampling for the Marine Microbes project, conducted by Bioplatforms Australia (BPA) [42]. Water samples were also collected from Towra Point as part of the NSW Food Authority’s Shellfish Safety program for the purpose of identification and enumeration of phytoplankton. A phytoplankton net was towed to collect a dense sample to verify species identity by microscopy. Lugol’s iodine (elemental iodine (5%) and potassium iodide (KI, 10%) and distilled water used at 1 mL/50 mL sample) was added immediately after collection to preserve cells [43]. In the laboratory, gravity-assisted membrane filtration was used to concentrate samples, and cell counts were completed using a Sedgewick Rafter counting chamber following a previously published protocol [43,44,45]. Highly toxic species were counted to a minimum level of detection of 50 cells^−L^, while others, including *P. minimum*, were counted to a minimum level of detection of 500 cells^−L^. All counts were completed using Zeiss Axiolab or Zeiss Standard microscopes with a maximum magnification of 1000×. All cells were identified to the nearest taxon able to be accurately identified, and if separation of species was not possible, the cells were assigned to a species group. 

### 2.2. Sampling, DNA Extraction for Amplicon Sequencing, Metabarcoding, and Physico-Chemical Data

All samples followed standard operating procedures (SOPs) outlined in Sections 1.5.1 and 1.7 of the Australian Microbiome Scientific Manual (version 2020) [46]. Briefly, 2 L samples were filtered in triplicate through 0.22 µm dia. pore-size polyethersulfone filters (Sterivex™, Merck KGaA, Darmstadt, Germany) to concentrate algal and bacterial cells. DNA was extracted using the QIAGEN DNeasy PowerSoil Kit according to the manufacturer’s instructions (QIAGEN, Hilden, Germany). Amplicon sequencing was completed using the HTS Illumina MiSeq technology at the Ramaciotti Centre for Genomics, University of New South Wales (UNSW). Samples were collected fortnightly at Towra Point and monthly at Bare Island. A series of 4× samples were collected during and after a rainfall event at both sites from 3 to 14 June 2016. 

### 2.3. Cell Culture and Culturing Conditions

Several 600 mL water samples were collected from Berowra Creek (−33.5342, 151.1459), a tributary of the Hawkesbury River in NSW, about 50 km north of Botany Bay, on 12 April 2019 following reports of a bloom of this species on 8 April 2019. The bloom was reported by Ana Rubio from Hornsby Shire Council, who regularly monitors and samples Berowra Creek. Clonal cultures of cells with the morphological features typical of *P. minimum* were isolated under a light microscope using a micropipette and cultured in a 96-well plate format. The Berowra Creek sample was successful for the isolation of *P. minimum*, and one isolate survived and was successively inoculated into 50 mL of media. The clonal isolate was grown in K media [47] in an incubator at 18 °C at a salinity of 35 PSU under a 12/12 h light cycle (±100 μmol photon m^−2^ s^−1^). A salinity of 35 PSU closely matches the salinity found at Berowra Creek where the strain was isolated from. A cultured strain of a closely related species, *Prorocentrum* cf. *balticum* (obtained from the Cawthron Institute Culture Collection, Nelson, New Zealand, CICCM, CAWD38), was used as a negative control in the qPCR assay. *P.* cf. *balticum* was maintained in culture in identical conditions to *P. minimum* in K media [47]. *Prorocentrum lima* (SM43), *Prorocentrum concavum* (SM46), and *Prorocentrum cassubicum* (CS881, from the Australian National Algal Culture Collection) cultures were grown in F/10 media [48] under a 12/12 h light cycle. *P. concavum* and *P. lima* were incubated at 28 °C, and *P. cassubicum* at 25 °C. 

### 2.4. Toxin Analysis

To prepare samples for toxin analysis using liquid chromatography–mass spectrometry (LC–MS, ThermoFisher Scientific Q Exactive, Waltham, MA, USA), 20 mL of a dense (28,000 cells mL^−1^) culture was centrifuged at 4000× *g* for 10 min to form a pellet, and the supernatant was discarded. The sample was then freeze-dried and stored at −20 °C. Analysis of TTX presence in the culture was completed by Dr. Chowdhury Sarowar at the Sydney Institute of Marine Science (SIMS) following an adapted method from [49]. 

Briefly, 5 mL of 1 mM acetic acid was added to the sample and vortexed for 90 s, after which the samples were placed in a boiling water bath for 5 min and then cooled to room temperature. The cooled sample was placed in an ultrasonic bath for 1 min and then centrifuged to pellet the cellular debris, and the supernatant was used with or without dilution for LC–MS analysis. A Thermo Scientific™ Q EXACTIVE™ MS (Waltham, MA, USA) was used for the detection of TTX. The source parameters for detection were as follows: sheath gas and auxiliary gas flow rates of 50 and 13, respectively (arbitrary units); a spray voltage of 3.5 kV; a capillary temperature of 263 °C; and an auxiliary gas heater temperature of 425 °C. 

Chromatographic separation was performed on a Thermo Scientific™ ACCELA™ UPLC system (Waltham, MA, USA). Analysis was performed using an Acquity UPLC BEH Phenyl 1.7 µm 100 × 2.1 mm column with an injection volume of 5 µL. The mobile phases used were A (water/formic acid/NH4OH at 500:0.075:0.3 v/v/v) and B (acetonitrile/water/formic acid at 700:300:0.1 v/v/v). Initial condition starts at A/B 2:98 at a flow rate of 400 µL/min and held for 5 min. The condition was then linearly changed for over 3.5 min from A/B (2:98) to A/B (50:50). The flow rate was then changed from 400 µL/min to 500 µL/min for over 2 min. The chromatographic condition was then rapidly changed to initial buffer conditions A/B (2:98) for over 0.5 min, while the flow rate was kept at 500 µL/min. The flow rate was then increased to 800 µL/min for over 0.5 min and held for 0.6 min. The flow rate was then decreased back to the initial flow rate of 400 µl/min, and the condition changed to A/B 100:0. A certified standard solution of TTX was sourced from Enzo Life Sciences (Exeter, UK). 

### 2.5. DNA Extraction and PCR for Strain Identification

Cell density was determined by enumeration with Lugol’s iodine-stained cells using a Sedgewick Rafter counting chamber [44,45]. Following microscopic counts, samples of the *Prorocentrum* spp. cultures were harvested by centrifugation at 4000× *g* for 10 min to be used for DNA extraction. DNA was extracted from the *P. minimum* (and other *Prorocentrum* spp. to be used for negative controls) cell pellets using the QIAGEN DNeasy PowerSoil Kit (QIAGEN, Hilden, Germany) according to the manufacturer’s protocol. The samples were eluted in 100 µL of buffer and stored at −20 °C until analysis. The quantity and quality of the extracted DNA were measured with a NanoDrop spectrophotometer (ThermoFisher Scientific, Waltham, MA, USA). Following DNA extraction, PCR amplification was completed using appropriate primers for the LSU rRNA and ITS rRNA regions. PCR amplification was conducted using the Bio-Rad T100 Thermal Cycler [50] (Bio-Rad Laboratories Inc., Hercules, CA, USA), targeting two rRNA regions. The LSU rRNA region was run using d1F (F) and d3B (R) primers, and the ITS rRNA region was run with ITSfwd (F) and ITSrev (R) primers (Table 1). PCR was run in 25 µL reactions with 12.5 µL of ImmoMix (Bioline, Sydney, NSW, Australia), 1 µL of BSA (bovine serum albumin), 7.5 µL of sterile water, 1.5 µL of forward primer (10 µM), and 1.5 µL of reverse primer (10 µM). The protocol used for PCR was 10 min at 95 °C, followed by 35 cycles of 95 °C for 20 s, 57 °C for 30 s, and 72 °C for 1 min, then held at 72 °C for 7 min. DNA fragments were cleaned using the DNA Clean and Concentrator (Zymo Research, Irvine, CA, USA) according to the manufacturer’s protocol and sequenced at Macrogen (Seoul, Korea). Contigs were formed using the sequences in Geneious Prime (v2019.2.1, Biomatters, Ltd., Auckland, NZ). Following the assembly of the contigs for each gene region, each sequence was uploaded to NCBI BLAST nucleotide sequence search to identify the strain and confirm that it was *P. minimum* (Table 2 and Table 3).

### 2.6. qPCR Assay Development 

#### 2.6.1. Primer Design

A published set of primers designed for a *P. minimum*-specific qPCR assay was tested [51]. Forward (F) and reverse (R) primers were designed to amplify a 325 bp region from the partial 18S rDNA sequence of *P. minimum* accessed from GenBank (AY421791.1) (Table 1). Twenty-two new sets of primers were designed after the above primer did not pass testing using the NCBI Primer-BLAST tool, targeting ITS regions 1 and 2 of partial sequence of the *P. minimum* strain CCMP698 (EU927537.1). The sizes of the qPCR products were from 70 to 130 bp in length, with the primers 20 to 25 bp in length, with the optimal T_m_ (melting temperature) set at 60 °C (Table 1). All sets were expected to be specific to the target sequence of *P. minimum*, as they were compared with all available sequences in the NCBI database and were unique to *P. minimum.* All 22 primer sets were run using an identical protocol (see qPCR Assays) with *P. minimum* DNA to determine the most sensitive and efficient assay. All valid primer sets were then subjected to specificity testing with other *Prorocentrum* spp. DNA. Primer sets that amplified other *Prorocentrum* spp. were disregarded, and then standard curve testing followed. 

#### 2.6.2. qPCR Assays 

qPCR was conducted using a 20 µL mix with 1 µL of DNA, 10 µL of Bio-Rad iTaq Universal SYBR Green Supermix (Bio-Rad Laboratories Inc., Hercules, CA, USA), 1 µL (at 10 µM concentration) of each of the forward and reverse primers, and 7 µL of sterile water. qPCR was performed with the following thermal cycling program of 95 °C for 10 min, followed by 40 cycles of 95 °C for 15 s and 60 °C for 1 min. A melt curve was performed on all runs, from 55 °C to 95 °C in 5 °C increments for 0.05 s each. All qPCR analyses and testing were run on the Bio-Rad CFX96 Touch Real-Time PCR Detection System in 96-well plates with a clear seal or clear plastic strips [52] (Bio-Rad Laboratories Inc., Hercules, CA, USA),). The previously published primers [51] and primer sets 1–12 followed the original qPCR protocol. Primers 13–22 were run with a modified protocol of 95 °C for 2 min, followed by 35–40 cycles of 95 °C for 15 s and 60 °C for 30 s. All assays were run using a generic thermal profile, as per above. The assays were tested for sensitivity using DNA extracted from the *P. minimum* cultures in duplicate and two negative controls containing no DNA (no template control (NTC)). Cross-reactivity of the primers was tested by running each assay on four other *Prorocentrum* spp. as negative controls. This step is crucial to ensure that the primers would only bind to *P. minimum* specifically. For other species, see Table 4. Unique primer sets developed using the NCBI Primer-BLAST tool were subjected to identical testing used for the published primer set [51]. Primer sets that were unable to amplify *P. minimum* were not sensitive (amplified past 35 cycles); those that amplified other *Prorocentrum* spp. at similar C_q_ values to *P. minimum* and those that had efficiencies outside 90–110% were disregarded. Those primer sets that passed specificity tests were then tested for their efficiency using a dilution series of gBlocks^®^ gene fragments (IDT, USA) of the ITS region and *P. minimum* DNA, both with known concentrations. Standard curves were created using a 10-fold dilution series over five different concentrations and plotted with the threshold cycle (C_q_) (*x*-axis) and natural log of concentration (cells/µL). The curves were used to calculate the efficiency (E) of the assay using $\;E=\;-1+10^{\left(-\frac{1}{slope}\right)}$. Efficiency of qPCR assays should fall between 90% and 110% [53]. This standard curve will also be used to quantify the amount of *P. minimum* cells in unknown concentrations from environmental samples [54,55]. The assay that had acceptable efficiency and specificity was used for analysing BPA samples. After the development of the assay, qPCR was run on all extracted DNA samples from Towra Point and Bare Island collected during the BPA project from 2016 to 2017 using only one of the primer sets that passed efficiency and specificity testing. If amplification was found in the no template control (NTC), a conservative cutoff value of 3.3 C_q_ points or more below the C_q_ of the NTC was set to accept the amplification of *P. minimum* in the samples. These data on the distribution and abundance of *P. minimum* were then compared with results obtained using light microscope identification and amplicon sequencing.

### 2.7. Bioinformatic Analysis 

Samples collected as part of the Marine Microbes project were subjected to amplicon sequencing using primer sets to target different organisms: eukaryotes, bacteria, archaea, and fungi (Table 1). The primer sets used targeted the V4 region of 18S rRNA (Table 1) found in all eukaryotes [56]. After sequencing, the reads were trimmed, merged, concatenated, and taxonomically classified using the Earth Microbiome Project (EMP) protocol [57,58]. Following this, the resulting actual sequence variants (ASUs) were assigned to taxonomic lineages and species using the PR2 taxonomic database (version 4.12) [59] with the DADA2 (version 1.16.0) [60] assignTaxonomy and assignSpecies functions in R. The resulting data were used to extract the occurrence and relative abundance of *P. minimum* in the sequence samples; these data were used to compare with the qPCR results. The classified data were also used to discover phytoplankton species that co-occur with *P. minimum.* The relative abundance of *P. minimum* was multiplied by 100 (1 µL was used for amplicon sequencing) to give the approximate abundance in the 2 L original sample and then divided by 2 to give cells L^−1^ to be able to compare with microscope count and qPCR abundance data. Data are submitted as Supplementary Material, Table S2: Towra Point ASVs and Table S3: Bare Island ASVs.

### 2.8. Environmental Parameters 

Physico-chemical data were collected during each sampling point according to the SOPs laid out in Australian Microbiome Scientific Manual Section 1.5. [46]. The physical parameters collected were temperature (°C), salinity (PSU), dissolved oxygen (% and mg/L), conductivity (s/m), total alkalinity (μmol/kg), and pH. The nutrients measured in the samples were nitrite, nitrate, oxidised nitrogen, phosphate, ammonium (all in µg/L), and total carbon dioxide (in μmol/kg). These data were statistically analysed with the qPCR *P. minimum* abundance data. 

### 2.9. Statistical Analysis

To test for relationships between *P. minimum* and environmental variables, the data were first checked for normality using the Shapiro–Wilks test due to the small dataset. After failing normality (*p* < 0.05), all variables were log-transformed and tested again for normality. Several variables remained non-normally distributed (*p* < 0.05), so a nonparametric testing approach was applied. The two sites were not found to show any significant differences (*p* > 0.05), so data were pooled for both sites for further analysis. Due to the disparate nature of the dataset, multiple regression was deemed inappropriate. Instead, Kendall’s tau-b correlation was used as it is more suitable for nonparametric small datasets and is more robust to error [61]. It was found to be a suitable analysis to determine preliminary relationships that could be investigated with further higher temporal data. A two-tailed correlation was run between all variables to assess the correlation between *P. minimum* abundance and environmental variables. Analyses were run in SPSS (v.26, IBM Corp, Armonk, NY, USA). 

To determine whether there were any significant correlations between *P. minimum* and other phytoplankton species, co-occurrence analysis was run using the probabilistic model developed by Veech (2013), which is included in the R package “cooccur” [62,63]. This method tests all possible pairwise associations between species across samples/sites, and the output is the probability that two species co-occur at a level that is more or less frequent than the observed frequency of co-occurrence [64]. The output provides information specific to *P. minimum* and its associations, as well as the number of random and significant associations between all species in the dataset. The amplicon sequencing output was used to create a presence/absence matrix with all phytoplankton species across all the BPA sampling dates to use in the analysis.

## 3. Results

### 3.1. Strain Isolation, Identification, and Toxin Testing

A strain of *P. minimum* was successfully isolated from a water sample from Berowra Creek, NSW, in April 2019. It was initially identified as *P. minimum* based on light microscopy. The strain was also identified as *P. minimum* based on sequencing of rRNA barcoding regions, as sequencing of the LSU (GenBank accession number MT856373) and ITS rRNA regions (GenBank accession number MT895109) matched eight different *P. minimum* strains (>99.5%) when queried against the NCBI nonredundant database using blastn (Table 3 and Table 4). The strain has been submitted to the Cawthron Institute Culture Collection (http://cultures.cawthron.org.nz/ (accessed on 12 February 2021)) as strain number CAWD359. The strain was tested for the presence of tetrodotoxin using a TTX standard. No TTX was detected, while TTX was detected in the spiked positive control. 

### 3.2. qPCR Assay Development and Testing

**Figure 2:**
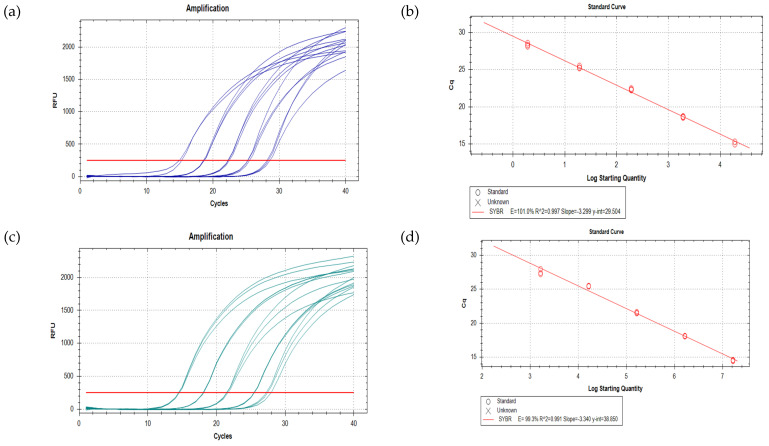
(**a**) Amplification plot showing dilution series using *P. minimum* culture DNA using primer set 20 and (**b**) DNA standard curve with *R^2^* value = 0.997 and E = 101%. (**c**) Amplification plot showing dilution series using *P. minimum* gBlocks using primer set 20 and (**d**) gBlock standard curve with *R*^2^ value = 0.991 and *E* = 99.3%.

A previously published assay with specific primers for *P. minimum* [51] was tested. This assay did not pass initial testing due to the amplification of other *Prorocentrum* species tested: *P.* cf. *balticum, P. lima, P. cassubicum*, and *P. concavum*. The assay was also not able to distinguish between products in the melt curve analysis (Table 5). The assay had a low efficiency of 70%. Following this, 22 new primer sets were tested (Table 5) to find one that was specific to *P. minimum*, sensitive, and efficient (90% < E < 110%). Only one primer set (20, Pmin 20F and Pmin 20R) displayed acceptable specificity and efficiency: E = 101% for *P. minimum* standard curve using DNA extracted from our strain (from 1.91 × 104 to 1.91 × 100 cells, Figure 2) and E = 99.3% for the standard curve using the gBlock synthetic gene fragment of the ITS region of *P. minimum* (from 1.64 × 10^7^ to 1.64 × 10^3^ copies, Figure 2) at an annealing temperature of 60 °C. Although this primer set was found to amplify other *Prorocentrum* species, this amplification occurred at similar or higher Cq values than that of the lowest *P. minimum* dilution point, which was the DNA equivalent of ~1.9 cells of *P. minimum*. This was even though all samples contained the DNA equivalent of >10^4^ cells of that *Prorocentrum* species. We considered this to be an acceptable level of cross-reactivity. The assay had a reliable detection limit of ~13 cells L^−1^ when only values of at least 3.3 Cq points or less than the NTC were taken into consideration [65,66]. Primer set 20 was then used to analyse the abundance of *P. minimum* in environmental samples collected from Botany Bay.

### 3.3. Comparison of qPCR, Light Microscope Count, and Amplicon Sequencing Abundance Results

**Figure 3:**
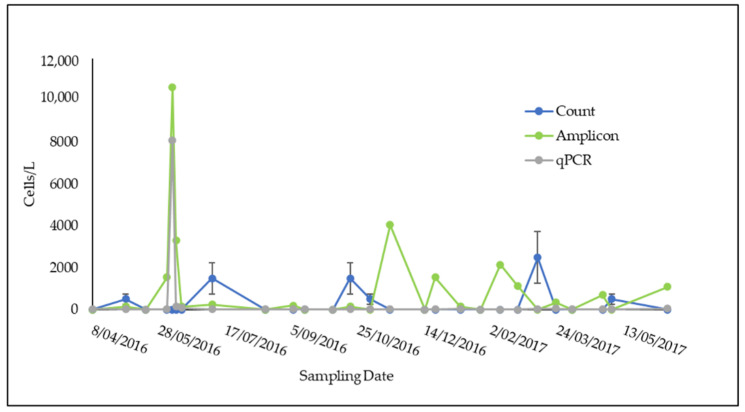
Towra Point *P. minimum* cell counts L^−1^ with amplicon sequencing, qPCR, and microscope counts across the BPA sampling period. Standard error bars are included for qPCR (too small to detect) and count data.

**Figure 4:**
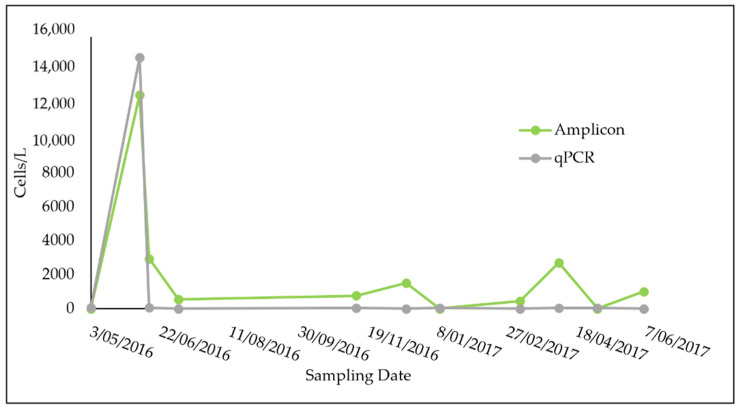
Bare Island *P. minimum* cell counts L^−1^ with amplicon sequencing and qPCR across the BPA sampling period. Standard error bars are included for qPCR (too small to detect).

During 2016–2017, *P. minimum* was recorded using the qPCR assay on 21 out of 27 sampling dates for Towra Point and 7 out of 17 dates for Bare Island, the two sites in Botany Bay (Figure 3 and Figure 4). Abundances varied from 0 cells L^−1^ to 8100 cells L^−1^ at Towra Point and from 0 cells L^−1^ to 14,800 L^−1^ at Bare Island. The highest peaks were recorded in early June for Towra Point and Bare Island (Figure 3 and Figure 4). No *P. minimum* was found on the 9 sampling dates between July 2016 and October 2016 at Bare Island (Figure 4). 

The estimates of the cell abundances of *P. minimum* using all three methods showed results that were of the same order of magnitude and often relatively similar (Figure 3 and Figure 4). The estimates of the abundance of *P. minimum* based on amplicon sequencing appeared to be consistently higher when compared with the qPCR and microscope count data (Figure 3). The highest abundances of *P. minimum* were found in June 2016; however, no microscope counts were completed for this month, so these data points were excluded in the following comparisons (Figure 3 and Figure 4). 

A highly significant relationship (*p* = 1.61 × 10^−14^/*p* = 0.00, Table 6) was found between the *P. minimum* abundance estimates derived from amplicon sequencing data and the *P. minimum*-specific qPCR, while the relationships between microscope counts and qPCR and microscope counts and amplicon sequencing were not significant (*p* > 0.05, Table 6). It is likely that the higher standard deviation in the method used for the light microscope cell count (Figure 3) may have led to the apparent differences in the cell counts of *P. minimum* using this method compared with that of the two molecular genetic methods.

### 3.4. Amplicon Sequencing Results

**Figure 5:**
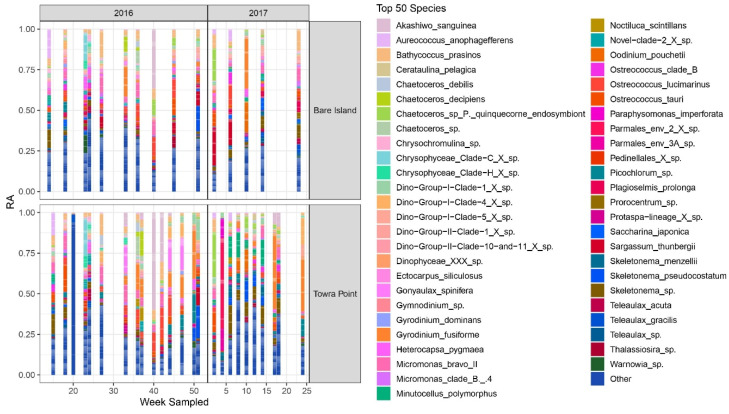
Relative abundance of the top 50 phytoplankton spp. at the Bare Island and Towra Point time series stations from April 2016 to June 2017. ASVs were assigned to phytoplankton species in the PR2.12 database. For clarity, only the top 50 by ASV abundance are shown. The less abundant taxa were lumped into the category “Other,” while those without taxa assignments to the genus level were omitted.

Using the amplicon sequencing method, 644 different phytoplankton ASVs in 428 genera were identified from the 27 samples from Towra Point, and 623 ASVs in 419 genera were identified from the 17 samples from Bare Island (Figure 5).

### 3.5. Factors Influencing the Growth of P. minimum in Botany Bay

The abundance of *P. minimum* in Botany Bay was found to be significantly correlated to the environmental variables, total dissolved CO_2_ (R = 0.34, *p* = 0.008), and salinity (R = −0.28, *p* = 0.035). All other variables were not found to be significantly correlated to *P. minimum* abundance. Data are submitted as Supplementary Material, S1: Physicochemical Data.

#### Co-occurrence Analysis

At Bare Island, *P. minimum* was found to have positive significant co-occurrence (*p* < 0.05) with four other phytoplankton species, *Pyramimonas gelidicola*, *Alexandrium pacificum*, *Euglyphida* sp., and *Goniomondales* sp. At Towra Point, *P. minimum* was found to have significant negative co-occurrence (*p* < 0.05) with two other phytoplankton species, *Prymnesiophyceae Clade F* sp. and *Blidingia dawsonii*, and positive significant associations (*p* < 0.05) with *Psammodictyon* sp., *Surirella* sp., *Tryblionella apiculata*, *Vampyrellida* sp., *Actinocyclus curvatulus*, *Cryothecomonas* sp., *Dino-Group-II-Clade-26* sp., *Massiteriidae* sp., *Navicula cryptocephala*, and *Navicula gregaria.*

## 4. Discussion

*Prorocentrum minimum* is a marine dinoflagellate that commonly occurs throughout the world and can form HABs [1,7,10]. HABs due to this species often occur in estuarine and coastal waters where aquaculture takes place, and in relation to that, death of shellfish has been reported [1,10]. *P. minimum* has been reported to show physiological flexibility with a global distribution across a range of conditions from temperate to subtropical [1,7,67]. It has been reported to produce TTX, a harmful neurotoxin [19,68,69,70]. Due to the potential harmful impacts of *P. minimum* on shellfish aquaculture, in this study, we aimed to develop new methods of investigating this species and apply them to environmental samples. In this study, a new culture of *P. minimum* was successfully isolated from Berowra Creek, Hawkesbury River, Australia. *P. minimum* has been linked to the production of TTX after a bloom in Vistonikos Bay, Greece, was positively correlated with TTX [27]. TTX was not detected in our strain. Genetic variability among strains may influence the toxicity of *P. minimum,* as well as the environmental conditions under which it is grown [1,24,26]. Due to the reported variability in toxicity in this species, future studies will be required to evaluate the toxicity of strains of *P. minimum*. As the alga is now successfully in culture with the Cawthron Institute Culture Collection, it allows future studies to look at more in depth toxin profiles, including how different environmental stressors and relationships with other known toxic algae or bacteria influence its toxicity.

qPCR assays have been developed over the past ~15 years for the detection and monitoring of HAB species [51,71,72]. qPCR has advantages over traditional light microscopy methods in that it is sensitive and rapid and allows for possible future automation. In the development of qPCR assays for the detection and quantification of specific taxa in environmental DNA, the most important considerations are the specificity of the assay in that it amplifies only the species of interest, and the amplification efficiency of assays with an efficiency of less than 90% will not give quantitative results across its full detection range [53,73]. Assays with an efficiency greater than 110% are considered to show significant inhibition to PCR amplification [74]. Amplification greater than 100% can be due to contamination in the sample, pipetting errors, inaccurate dilution series, and primer dimers [29]. For this study, a previously published qPCR assay developed for *P. minimum* was originally tested [51], which targeted a fragment of the small subunit ribosomal (SSU/18S) RNA gene. However, it was found to amplify several other nontarget *Prorocentrum* spp. and have a low efficiency (Table 5, 70%). Therefore, new primer sets were designed to develop a new qPCR assay for *P. minimum* with the aim of being specific, sensitive, and efficient. Twenty-two unique primer sets were designed and tested with variable results (Table 5). Only one of the primer sets was found to be sufficiently specific and efficient and was used to examine environmental samples for the presence of *P. minimum* (primer set 20, Table 5). The newly designed assay was based on the ITS rDNA gene region, which is more variable and faster evolving than the SSU rDNA gene among dinoflagellate species [75,76]. The assay did have a low level of cross-reactivity with the most genetically similar species, *P.* cf. *balticum.* However, *P.* cf. *balticum* could be distinguished from *P. minimum* due to a higher temperature on the melt curve profile. Several studies have used melt curve differences to discriminate similar species [77,78]. When analysed for efficiency, the new primer set showed E = 101% (*P. minimum* DNA) and E = 99% (gBlock synthetic DNA) (Table 5). The new assay amplifies a much shorter fragment than the previously published assay (71 bp compared with 325 bp), and this may account for its greater amplification efficiency [79]. The qPCR assay developed for *P. minimum* is more sensitive than most light microscopy counting methods, with a reliable detection limit of 13 cells L^−1^ [65,66].

Molecular barcoding, or amplicon sequencing, which involves PCR amplification of environmental DNA (eDNA) and then sequencing of short (~600) [80] “barcoding” gene regions using high-throughput sequencing (HTS), is another molecular genetic method that has begun to be used in phytoplankton research [81,82,83]. Amplicon sequencing uses broad-range primers designed to amplify conserved regions across whole domains of life—in this case, eukaryotes [56,84]. A major problem with the use of amplicon sequencing as a tool for quantifying microbial eukaryotes is that the “barcoding” genes may be present in multiple copies that can be variable among microalgal populations and species, meaning that sequenced gene amplicons may not reflect the true abundance of a species in the sample. qPCR is not immune to this problem; however, the impact is minimised by designing primers that amplify gene regions only present in a specific species and using a standard curve with known amounts of target.

However, in this study, the sequencing of amplicons of eukaryotic V4 regions of SSU rDNA from samples from Botany Bay did not show a significantly different quantity of *P. minimum* compared with the quantification based on qPCR (Figure 3 and Figure 4 and Table 6). In addition, the results of this method have shown a previously unknown level of phytoplankton diversity in Botany Bay, detecting over 600 eukaryotic microbial ASVs between the two sites in Botany Bay. Previously, phytoplankton identification using light microscopy had led to the detection of only ~100 species or fewer in 10 years of phytoplankton monitoring at Botany Bay [85,86]. In this study, only 43% of all phytoplankton ASVs were able to be classified to species level using the 18S V4 primer set and the PR2 database [59]. Further development of reference databases of 18S V4 sequences from reference “voucher” specimen taxa curated by taxonomists is an important factor in the future of HTS to enable a more complete and accurate picture of microbiome species composition [87,88]. Another possible approach that may lead to a more specific identification of taxa is the use of other primer pairs that amplify other amplicon regions, such as the LSU rDNA region in dinoflagellates, the SSU (16S) plastid genes, CO1, cytochrome *b*, or other mitochondrial gene regions [82,83,89,90]. In previous studies, it was found that some groups of taxa, such as dinoflagellates, can be identified more readily using LSU rDNA regions, rather than SSU rDNA [83].

The collection and preservation of water samples for the identification and manual counting of cells with light microscopy has been the “gold standard” method used to study phytoplankton abundances [91,92,93]. The accuracy of light microscope-based microalgal enumeration is highly variable depending on the particular technique chosen, the counting effort, and the taxonomic expertise of the technician [81]. Compared with light microscopy enumeration, amplicon sequencing has been shown to be extremely sensitive and has the capacity to identify all phytoplankton species in a sample without requiring any taxonomic expertise. qPCR is an optimal technique for the enumeration of a particular target species, as the limit of detection is low, and the accuracy of the method is independent of the effort or taxonomic skills of the operator. It is relatively inexpensive, rapid, sensitive, and specific and, therefore, is highly suited to adoption for ongoing monitoring programs. qPCR can also be completed in situ at the time of sampling to get rapid results and can be used by trained shellfish producers to get results of HABs on-site.

The light microscopy counting method utilised in this study had a larger-than-average error rate, a high detection threshold of *P. minimum* (500 cells L^−1^ compared with 13 cells L^−1^ with qPCR), and comparatively fewer data points when compared with the molecular methods. Adoption of other light microscope counting methods, like the Utermöhl counting chamber [94], and the use of a DNA-based stain (i.e., a fluorescence in situ hybridisation (FISH) probe [95]) may have led to more accurate assessments of the abundance of *P. minimum* and the detection of cryptic species. For research into HAB ecology, a combination of the use of amplicon sequencing, to first determine the diversity of phytoplankton, particularly cryptic and small species, and then qPCR, to quantify the exact cell abundance, would appear to give optimal information for ecological inference and understanding of co-occurrence patterns. This two-step molecular pathway appears to be the most appropriate method for future development [29,35,71,92,96].

Botany Bay is an estuary in southeast Australia that is extensively modified [43], containing an international shipping port (Port Botany), an oil fuelling station, recreational beaches, industrial estates, and urban developments [97]. The bay is a highly populated area and is impacted by freshwater flows from the Georges and Cooks Rivers, both also extensively modified and surrounded with urban developments [97]. Despite the modification, Botany Bay is also home to a Ramsar wetland and one remaining oyster farm, both at Towra Point [98]. Thus, it is an important site for ongoing monitoring of HAB species, as they can impact not only the shellfish production but also the quality of the water for recreational and industrial purposes. Botany Bay and the Georges River have both previously experienced HABs, including *Noctiluca scintillans*, *Alexandrium pacificum*, other *Alexandrium* spp., and *Heterocapsa* spp. [99].

Two sites in Botany Bay were sampled from April 2016 to June 2017: Bare Island and Towra Point (Figure 1). Due to the extensively modified nature of the bay and its surroundings, and the nutrient input that can occur in relation to this land runoff, it was expected that *P. minimum* may be abundant at these sites. It was also expected that *P. minimum* may be particularly high in abundance at Towra Point, which is impacted by freshwater flows, as this species has been shown to flourish in low salinities with high nutrient freshwater inputs [1,70]. *P. minimum* was found to be in low abundance for most of the sampling period at both sites, detected at ~30 cells L^−1^ at both sites for most of the year of sampling (Figure 3 and Figure 4). There was one peak in the abundance of *P. minimum* (8000–14,000 cells L^−1^) at both sites, on 7 June 2016 (Figure 3 and Figure 4). This could still be considered a low value for *P. minimum,* which has been detected at “bloom” levels upwards of 10 million cells^−L^ [11]. The low presence of *P. minimum* is an important current baseline for monitoring the health of Botany Bay and other southeast Australian estuaries.

The abundance of *P. minimum* was found to have a significant positive relationship with total CO_2_, contrary to a previous finding that found that increased CO_2_ had no relationship with the abundance of *P. minimum* [100]. *P. minimum* was also found to have a weak but significant negative relationship with salinity, which supports previous findings that *P. minimum* grows preferentially in decreasing salinities [1,70,101]. *P. minimum* was not found to have a significant relationship with any other environmental variables; however, it is likely that there may be a time lag between an environmental change and increase or decrease in *P. minimum* [102,103]. Incorporating a measure of exposure of *P. minimum* to environmental variables would require a higher temporal sampling frequency than what was undertaken in the present study. However, the correlations we found (+ve CO2 and −ve salinity) between *P. minimum* and the environmental variables measured are hypothesis forming and should be further investigated in Australian waters.

An analysis of phytoplankton species that significantly co-occurred with *P. minimum* in Botany Bay is useful, as in the past, toxicity attributed to blooms of *P. minimum* may have been also associated with the presence of *Dinophysis* spp., which are the main causative agents of diarrhetic shellfish poisoning (DSP), even when present in low abundances, such as ~100 cells L^−1^ [1,21,22,104]. Due to the potential uncertainties of amplicon sequencing-based estimates of the absolute abundance of cells in a sample, the data were analysed as a presence/absence matrix across all sampling dates [29,37]. *P. minimum* at Towra Point was found to significantly co-occur with 12 other phytoplankton species and at Bare Island with 4 other phytoplankton species. Of all the co-occurring species, only 1 is a toxin-producing species, *Alexandrium pacificum. A. pacificum* is an important HAB species due to the severity of bloom impacts in Australia, New Zealand, Korea, Japan, and other countries [72,105,106,107]. *A. pacificum* produces paralytic shellfish toxins (PSTs). *P. minimum* blooms have previously been associated with symptoms characteristic of PSTs [68,69]; however, there is still a possibility that it can produce other toxins not yet classified [19,20].

## 5. Conclusions

A sensitive, specific, and efficient *P. minimum* qPCR assay was successfully developed and will allow for high-throughput information to be collected on the distribution and abundance of this species. A new strain of *P. minimum* was also isolated from Berowra Creek, NSW, and shown to not produce tetrodotoxin. *P. minimum* was found to generally be in a low abundance in Botany Bay across all seasons during the BPA Marine Microbes sampling period (April 2016–June 2017), with one peak in its abundance at Towra Point and Bare Island in June. *P. minimum* was found to be significantly correlated to total CO_2_ and to a decrease in salinity at the sites in Botany Bay. Further field and laboratory studies may be useful to determine more detailed information on the environmental variables associated with blooms of *P. minimum* in southeast Australia. *P. minimum* was found to positively co-occur with *A. pacificum*, which produces PSTs. This association may be relevant to the management of harmful algal blooms in Botany Bay and other oyster-producing estuaries in southeast Australia. qPCR is a useful method for the monitoring of particular HAB species as it is rapid, specific, sensitive, and efficient, while the use of amplicon sequencing based on the V4 region of the 18S rDNA found a level of microbial eukaryotic species diversity that was approximately six times greater than that previously known from this site. In the future, these two methods may be combined as a valuable tool for HAB research in Australian waters.

## Figures and Tables

**Table 1:** Names and sequences of all the primers used in this project. Primers 200F and 525R are taken from [51]. The other primers were designed using the online Primer-BLAST software (NCBI). All reverse primers are in reverse complement.

**Primer Sequences for qPCR**			
Name	Sequence (Forward)	Name	Sequence (Reverse)
Pm 200F	TGTGTTTATTAGTTACAGAACCAGC	Pm 525R	AATTCTACTCATTCCAATTACAAGACAAT
1F Pmin	CGCAGCGAAGTGTGATAAGC	1R Pmin	TCTGGAAAGGCCAGAAGCTG
2F Pmin	TCGGCTCGAACAACGATGAA	2R Pmin	AAGCGTTCTGGAAAGGCCAG
3F Pmin	TTCTGGCCTTTCCAGAACGC	3R Pmin	CATGCCCAACAACAAGGCAA
4F Pmin	CGTATACTGCGCTTTCGGGA	4R Pmin	CACACAGAAACACACAAGCGT
5F Pmin	CCTTTCCAGAACGCTTGTGTG	5R Pmin	CTGGGCACTAGACAGCAAGG
6F Pmin	CAGGCTCAGACCGTCTTCTG	6R Pmin	AGCGTTCTGGAAAGGCCAG
7F Pmin	CAACAGTTGGTGAGGCTCT	7R Pmin	ATTCAAAAACACAGAAGATCAGGAA
8F Pmin	AACAACAGTTGGTGAGGCTCTG	8R Pmin	CAAAAACACAGAAGATCAGGAAGAC
9F Pmin	GTGAGGCTCTGGGTGGG	9R Pmin	CAAAAACACAGAAGATCAGGAAGAC
10F Pmin	TCATTCGCACGCATCCATTC	10R Pmin	AAGGACAGGCACAGAAGACG
11F Pmin	TTCAGTGCACAGGGTCTTCC	11R Pmin	GTCTTGGTAGGAGTGCGCTG
12F Pmin	GCCTTTCCAGAACGCTTGTGT	12R Pmin	GCTGACCTAACTTCATGTCTTGG
13F Pmin	CGCTTGTGTGTTTCTGTGTG	13R Pmin	CCATGCCCAACAACAAGGC
14F Pmin	TCTTCCCACGCAAGCAACT	14R Pmin	CGGGTTTGCTGACCTAAACT
15F Pmin	ACATTCGCACGCATCCATTC	15R Pmin	TTGCTGCCCTTGAGTCTCTG
16F Pmin	AACAGTTGGTGAGGCTCTGG	16R Pmin	AAGGACAGGCACAGAAGACG
17F Pmin	ACAACAGTTGGTGAGGCTCT	17R Pmin	TTGCTGCCCTTGAGTCTCTG
18F Pmin	CAGTTGGTGAGGCTCTGGG	18R Pmin	CAGAAGACGGTCTGAGCCTG
19F Pmin	TTCAGTGCACAGGGTCTTCC	19R Pmin	CATGCCCAACAACAAGGCAA
20F Pmin	ATTCCAGCTTCTGGCCTGTC	20R Pmin	TAGTTGCTTGCGTGGGAAGA
21F Pmin	CTGTCCAGAACGCTTGTGTG	21R Pmin	CTTCTAGTTGCTTGCGTGGG
22F Pmin	TTCCCACGCAAGCAACTAGA	22R Pmin	GCACTAGACAGCAAGGCCA
**Primer Sequences for Amplicon Sequencing**			
ILM_Euk_1391f Forward	AATGATACGGCGACCACCGAGATCTACAC TATCGCCGTT CG GTACACACCGCCCGTC	ILM_EukBr Reverse	CAAGCAGAAGACGGCATACG GAT CA TGATCCTTCTGCAGGTTCACCTAC
**Primer Sequences for PCR and Sanger Sequencing**			
d1f	ACCCGCTGAATTTAAGCATA	d3b	TCGGAGGGAACCAGCTACTA
ITSfwd	TTCCGTAGGTGAACCTGCGG	ITSrev	ATATGCTTAAATTCAGCGGGT

**Table 2:** Top 5 BLAST nucleotide sequence hits of the LSU rRNA sequence of *Prorocentrum minimum* from Berowra Creek, CAWD359, as compared with sequences of *P. minimum* strains on the NCBI database, including linked accession numbers.

Strain Description	Percent Identity	Accession
*Prorocentrum minimum* strain DAB02 28S	99.66%	KU999985.1
*Prorocentrum minimum* strain D-127	99.66%	JX402086.1
*Prorocentrum minimum* isolate PIPV-1	99.54%	JQ616823.1
*Prorocentrum minimum* isolate SERC	99.54%	EU780639.1
*Prorocentrum minimum* strain Pmin1	99.54%	AY863004.1

**Table 3:** Top 5 BLAST nucleotide sequence hits of the ITS rRNA sequence of *P. minimum* from Berowra Creek, CAWD359, as compared with sequences of *P. minimum* strains on the NCBI database.

Strain Description	Percent Identity	Accession
*Prorocentrum minimum* strain D-127	99.67%	JX402086.1
*Prorocentrum minimum* strain AND3V	100.00%	EU244473.1
*Prorocentrum minimum* isolate PIPV-1	99.35%	JQ616823.1
*Prorocentrum minimum* strain PMDH01	99.35%	DQ054538.1
*Prorocentrum minimum* strain NMBjah049	99.67%	KY290717.1

**Table 4:** All species used as negative controls for specificity testing of the *P. minimum* qPCR assay. Species names and which culture collection they can be found are listed, as well as the strain ID.

Species Name	Culture Collection	Strain ID #
*Prorocentrum* cf. *balticum*	Cawthron Culture Collection (CICCM)	CAWD38
*Prorocentrum cassubicum*	Australian National Culture Collection (ANAAC)	CS-881
*Prorocentrum concavum*	Seafood Safety Team, University of Technology Sydney (UTS)	Pmona (SM46)
*Prorocentrum lima*	Seafood Safety Team, University of Technology, Sydney (UTS)	SM43

**Table 5:** Specificity and efficiency testing of each primer set including the previously published one [51]. (+) and (−) mean amplification or no amplification, respectively, and (/) denotes amplification at a high C_q_ and/or was at or below the lowest dilution point on the *P. minimum* standard curves and/or was amplified but had a different melt peak. N/A means the primer set was not subjected to that test.

Specificity						Efficiency	
Primer Set	*P. minimum*	*P.* cf. *balticum*	*P. cassubicum*	*P. concavum*	*P. lima*	gBlock (%)	*P. min* DNA (%)
Pm 200F/525R	+	+	−	/	/	70	−
3	+	/	−	−	−	65	−
4	+	+	−	−	−	64	−
5	+	+	+	+	−	62	−
6	+	+	+	+	+	65	−
7	+	/	−	−	−	57	−
8	+	+	−	−	−	56	−
9	+	+	−	−	−	58	−
10	+	+	N/A	/	/	60	−
11	+	−	N/A	−	−	76	−
12	+	/	N/A	/	−	85	−
13	+	−	+	N/A	+	43	−
15	+	+	N/A	+	+	328	335
19	+	+	N/A	+	+	220	383
20	+	/	−	/	/	99	101
21	+	+	+	+	+	N/A	N/A
22	+	+	+	+	+	115	147

**Table 6:** Linear regression between qPCR, metabarcoding, and light microscopy.

	qPCR vs. Amplicon Sequencing	qPCR vs. Count	Amplicon Sequencing vs. Count
Multiple R	0.90	0.18	0.23
R^2^	0.82	0.03	0.05
Adjusted R^2^	0.81	−0.02	0.00
Standard Error	1172.31	670.05	662.94
df	35	20	20
*p* (Significance)	0.00	0.43	0.31

## Data Availability

The data presented in this study are available in Supplementary Material; Table S1 for physicochemical data, Table S2 for Towra Point ASVs and Table S3 for Bare Island ASVs.

## Supplementary Materials

The following are available online at https://www.mdpi.com/2076-2607/9/3/510/s1, Table S1: Physicochemical data, Table S2: Towra Point ASV data, Table S3: Bare Island ASV data.
