# Correction: McLennan et al. Assessing the Use of Molecular Barcoding and qPCR for Investigating the Ecology of *Prorocentrum minimum* (Dinophyceae), a Harmful Algal Species. *Microorganisms* 2021, *9*, 510

**DOI:** 10.3390/microorganisms10101906

**Published:** 2022-09-26

**Authors:** Kate McLennan, Rendy Ruvindy, Martin Ostrowski, Shauna Murray

**Affiliations:** Faculty of Science, University of Technology Sydney, Ultimo, NSW 2007, Australia

The authors wish to make the following corrections to this paper [1].

A correction has been made to Table 1:

**Table 1:** Names and sequences of all the primers used in this project. Primers 200F and 525R are taken from [51]. The other primers were designed using the online Primer-BLAST software (NCBI). All reverse primers are in reverse complement.

Name	Sequence (Forward)	Name	Sequence (Reverse)
**Primer Sequences for qPCR**			
Pm 200F	TGTGTTTATTAGTTACAGAACCAGC	Pm 525R	AATTCTACTCATTCCAATTACAAGACAAT
1F Pmin	CGCAGCGAAGTGTGATAAGC	1R Pmin	TCTGGAAAGGCCAGAAGCTG
2F Pmin	TCGGCTCGAACAACGATGAA	2R Pmin	AAGCGTTCTGGAAAGGCCAG
3F Pmin	TTCTGGCCTTTCCAGAACGC	3R Pmin	CATGCCCAACAACAAGGCAA
4F Pmin	CGTATACTGCGCTTTCGGGA	4R Pmin	CACACAGAAACACACAAGCGT
5F Pmin	CCTTTCCAGAACGCTTGTGTG	5R Pmin	CTGGGCACTAGACAGCAAGG
6F Pmin	CAGGCTCAGACCGTCTTCTG	6R Pmin	AGCGTTCTGGAAAGGCCAG
7F Pmin	CAACAGTTGGTGAGGCTCT	7R Pmin	ATTCAAAAACACAGAAGATCAGGAA
8F Pmin	AACAACAGTTGGTGAGGCTCTG	8R Pmin	CAAAAACACAGAAGATCAGGAAGAC
9F Pmin	GTGAGGCTCTGGGTGGG	9R Pmin	CAAAAACACAGAAGATCAGGAAGAC
10F Pmin	TCATTCGCACGCATCCATTC	10R Pmin	AAGGACAGGCACAGAAGACG
11F Pmin	TTCAGTGCACAGGGTCTTCC	11R Pmin	GTCTTGGTAGGAGTGCGCTG
12F Pmin	GCCTTTCCAGAACGCTTGTGT	12R Pmin	GCTGACCTAACTTCATGTCTTGG
13F Pmin	CGCTTGTGTGTTTCTGTGTG	13R Pmin	CCATGCCCAACAACAAGGC
14F Pmin	TCTTCCCACGCAAGCAACT	14R Pmin	CGGGTTTGCTGACCTAAACT
15F Pmin	ACATTCGCACGCATCCATTC	15R Pmin	TTGCTGCCCTTGAGTCTCTG
16F Pmin	AACAGTTGGTGAGGCTCTGG	16R Pmin	AAGGACAGGCACAGAAGACG
17F Pmin	ACAACAGTTGGTGAGGCTCT	17R Pmin	TTGCTGCCCTTGAGTCTCTG
18F Pmin	CAGTTGGTGAGGCTCTGGG	18R Pmin	CAGAAGACGGTCTGAGCCTG
19F Pmin	TTCAGTGCACAGGGTCTTCC	19R Pmin	CATGCCCAACAACAAGGCAA
20F Pmin	ATTCCAGCTTCTGGCCTGTC	20R Pmin	TAGTTGCTTGCGTGGGAAGA
21F Pmin	CTGTCCAGAACGCTTGTGTG	21R Pmin	CTTCTAGTTGCTTGCGTGGG
22F Pmin	TTCCCACGCAAGCAACTAGA	22R Pmin	GCACTAGACAGCAAGGCCA
**Primer Sequences for Amplicon Sequencing**			
Modified TAReuk454FWD1	CCAGCASCYGCGGTAATTCC	Modified TAReukREV3	ACTTTCGTTCTTGATYRATGA
**Primer Sequences for PCR and Sanger Sequencing**			
d1f	ACCCGCTGAATTTAAGCATA	d3b	TCGGAGGGAACCAGCTACTA
ITSfwd	TTCCGTAGGTGAACCTGCGG	ITSrev	ATATGCTTAAATTCAGCGGGT

The authors apologize for any inconvenience caused and state that the scientific conclusions are unaffected. The original publication has also been updated.
